# A Structural Equation Model of Health-Related Quality of Life in Chinese Patients With Rheumatoid Arthritis

**DOI:** 10.3389/fpsyt.2021.716996

**Published:** 2021-08-04

**Authors:** Biyu Shen, Haoyang Chen, Dongliang Yang, Ogbolu Yolanda, Changrong Yuan, Aihua Du, Rong Xu, Yaqin Geng, Xin Chen, Huiling Li, Guang-Yin Xu

**Affiliations:** ^1^Center for Translational Pain Medicine, Institute of Neuroscience, Soochow University, Suzhou, China; ^2^Department of Nursing, Nursing School of Soochow University, Suzhou, China; ^3^Department of Nursing, Nursing School of University of Maryland, Baltimore, MD, United States; ^4^Department of Nursing, The Second Affiliated Hospital of Nantong University, Nantong, China; ^5^Department of Mathematics, Cangzhou Medical College, Cangzhou, China; ^6^Department of Nursing, Fudan University, Shanghai, China; ^7^Department of Rheumatology, Zhengzhou Gout and Rheumatology Hospital, Zhengzhou, China; ^8^Department of Rheumatology, The First Affiliated Hospital of Soochow University, Suzhou, China; ^9^Department of Rheumatology, The Second People's Hospital of Changzhou, Changzhou, China

**Keywords:** rheumatoid arthritis, quality of life, body image, pain, depression

## Abstract

**Background:** The aim of this study was to examine how body image, Disease Activity Score in 28 joints, the feeling of being anxious, depression, fatigue, quality of sleep, and pain influence the quality of life (QoL) in patients with rheumatoid arthritis (RA).

**Methods:** A multicenter cross-sectional survey with convenience sampling was conducted from March 2019 and December 2019, 603 patients with RA from five hospitals were evaluated using the Body Image Disturbance Questionnaire, Disease Activity Score in 28 joints, Hospital Anxiety and Depression Scale, Fatigue Severity Scale, Pittsburgh Sleep Quality Index, Short Form 36 Health Survey, and Global Pain Scale. The relationship between quality of life and other variables was evaluated by using the structural equation model (SEM).

**Results:** A total of 580 patients were recruited. SEM fitted the data very well with a root mean square error of approximation (RMSEA) of 0.072. Comparative fit index of 0.966, and Tucker-Lewis index of 0.936. The symptoms and the normalized factor load of six variables showed that the normalized factor load of pain was 0.99.

**Conclusions:** The QoL model was used to fit an SEM to systematically verify and analyze the population disease data, biological factors, and the direct and indirect effects of the symptom group on the QoL, and the interactions between the symptoms. Therefore, the diagnosis, treatment and rehabilitation of RA is a long-term, dynamic, and complex practical process. Patients' personal symptoms, needs, and experiences also vary greatly. Comprehensive assessment of patients' symptoms, needs, and experiences, as well as the role of social support cannot be ignored, which can help to meet patients' nursing needs, improve their mood and pain-based symptom management, and ultimately improve patients' QoL.

## Introduction

Rheumatoid arthritis (RA) is a chronic inflammatory joint disease that may cause cartilage and bone damage, as well as significant disability (1). RA usually involves the small joints in the hands and feet and is characterized by joint pain, joint swelling, and synovial joint destruction (2). Its prevalence in adults in the USA and Europe is ~0.42–1.25% and 0.28–0.45% in China (3, 4).

Individuals with RA frequently report reduced health-related quality of life (HRQoL), which is an indicator of the impact of one's health on his/her physical, emotional, and social well-being (5). RA is very painful and affects the social activities of the patient. Many patients have been sick since childhood, this painful experience may be associated with psychosocial impairment and may influence the negative outcomes in RA (6). Patients with RA show systemic symptoms, such as pain, stiffness, muscle weakness, fatigue, and joint swelling; all of these might cause irreversible destruction of joints or deformities accompanied by physical disability when the disease progresses; all of the abovementioned symptoms are the most common causes of continual pain and impaired functioning (7), and significantly decrease a person's mobility, productivity, and QoL (8, 9).

Pain is the symptom that the patient feels most directly. RA patients also have a higher incidence of depression (10). More than half of the patients experience fatigue. Fatigue is described as either physical or mental fatigue, which combined may lead to disability. Patients feel tired, depressed, or frustrated, and are unable to complete their daily tasks (11, 12). Fatigue had a substantial influence on the patients' lives, while pain was the dominant factor in the fatigue experience and degree (13). Poor sleep quality is common in patients with RA and may lead to disease aggravation and decreased HRQoL. The prevalence of body image disturbance (BID) is 24.2%, and poor social functioning and anxiety are also present (14). Besides, increasing disease severity has been associated with worsening disability, pain, fatigue, QoL, and work and activity impairment (15).

Moreover, RA is related to reverse characteristics in terms of demographics, clinical features, as well as psychological status. An uncertain future concerning physical ability, work and employment status, family responsibilities, and social activities can be difficult to face, especially in individuals with RA. RA patients are at higher risk of developing comorbidities (16), which are also associated with advanced age. In China, patients with RA have a similar prevalence of comorbidities when compared to those in other Asian countries. Advanced age and long disease duration are possible risk factors for comorbidities, which may increase mortality and affect treatment strategies, resulting in worse outcomes (17, 18). Patients with RA tend to have a higher risk for many comorbidities (19, 20).

Improving patients' symptoms can improve their quality of life. Depression is a major determinant of functional capacity in RA (21). Effective social support may relieve patients' fatigue, which is related to patients' disease activity and QoL (22).

Many studies have considered a simple associative relationship between two of the following variables or have evaluated the variables as a single item instead of one with multiple dimensions (23–25): pain, fatigue, depression, sleep quality, and QoL impact. There has been no systematic and comprehensive analysis of the relationship between patients' demographic data and symptoms, and the causal relationship between RA symptoms and QoL. It is possible to evaluate and manage patients comprehensively and provide a theoretical basis to formulate appropriate objective interventions to improve QoL.

However, improving the quality of life does not always accompany QoL improvement. Physical aspects are commonly evaluated as most important and are related to a patient's physical condition only as a consequence of illness and treatment.

**Figure 1:**
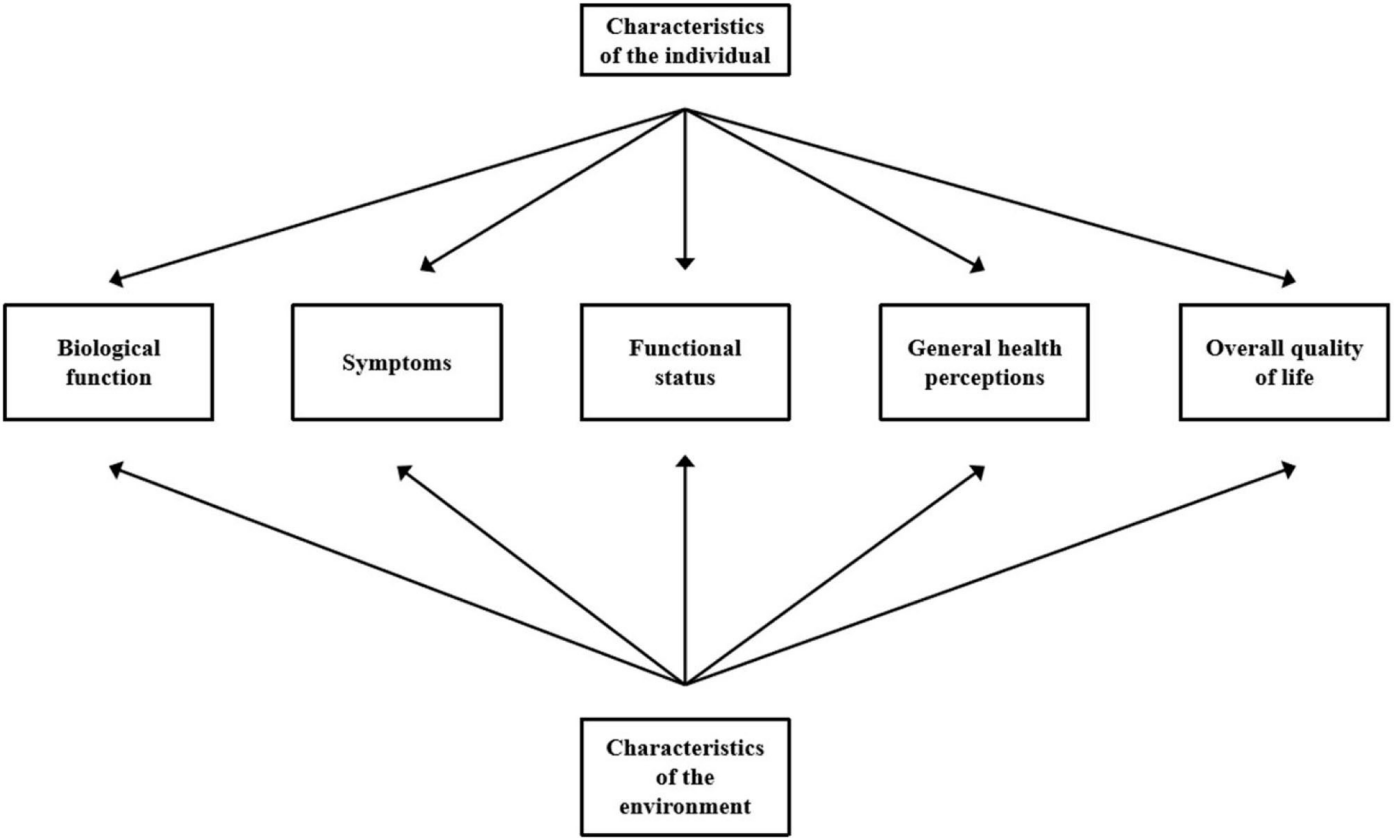
Revised Wilson and Cleary model of health-related quality of life. Adapted from “Linking Clinical Variables with Health-Related Quality of Life: A Conceptual Model of Patient Outcomes,” by Wilson and Cleary (26). Copyright by *JAMA*. Used with permission.

The framework of this study is derived from QoL research; the current challenge is to devise a model to clarify the elements of HRQoL and relationships among them. Wilson and Cleary have suggested specific causal relationships between health concepts encompassing biological, social, and psychological variables (Figure 1) (26).

A linear progression without dominant reciprocal effects or links between nonadjacent concepts has been proposed. Wilson and Cleary's model outlines potential causal relationships between the variables that play a major role in the origins of HRQoL.

Based on the above rationale, we aimed to: (1) describe the current status of HRQoL in patients with RA, including parameters such as gender socioeconomic status, and disease characteristics and explore their relevance; (2) perform an exploratory analysis of relevant parameters including the effects of pain, fatigue, and physical image disorders, depression, sleep quality, disease activity, HRQoL, and the interaction between multiple variables; (3) assess HRQoL and determine which factors, based on the Wilson and Cleary model, contribute to the prediction of HRQoL among patients with RA; and (4) estimate the potential impact of success on RA-HRQoL to provide a theoretical basis for effective interventions against identified factors.

## Materials and Methods

### Study Design and Patient Recruitment

A multicenter cross-sectional study with a convenience sampling method was conducted. Patients with RA were recruited from five hospitals between March 2019 and December 2019. The inclusion criteria included RA diagnosis according to the American Rheumatism Association 1987 revised criteria, age ≥18 years, able to interact in Chinese efficiently, and willing to provide written informed consent. People who have cognitive impairment or current severe diseases, such as cancer and stroke, were not included.

Altogether, 603 patients with RA were consecutively invited to participate in the cross-sectional study, and 580 (96.2%) were eventually included in the analysis. The Spearman correlation coefficient was used to determine the correlation between variables. The relationship between quality of life and other variables was evaluated by using the Structural Equation Model (SEM).

### Data Collection and Ethical Considerations

The study was conducted in accordance with the principles of the Declaration of Helsinki and approved by the ethics committee of Soochow University (SUDA20200225H11). All subjects meeting the criteria were asked to participate. Questionnaires were distributed by two well-trained researchers to the eligible participants. All participants were informed about (a) the aim and significance of this research, (b) confidentiality of patient data, and (c) that their engagement was totally voluntary, and they could withdraw from the research at any time. All data in the questionnaires that were completed were made confidential. A unique identification number was placed at the upper portion of the questionnaire.

### Measurement Tools

The Body Image Disturbance Questionnaire comprised the following seven items (27): recognized concern, preoccupation, avoidance of role, the emotional suffering of their own appearance, as well as appearance impairment in social, educational, and work function. The Cronbach's alpha value was 0.877.

The patient's disease activity was measured using the Disease Activity Score in 28 joints (DAS28) (28). Four aspects were included in this questionnaire, which were calculated by using a software program that measures 28 swollen joint counts, 28 tender joint counts, the rate of erythrocyte sedimentation rate, as well as the patient's recognition of disease activity from 0 mm (not active at all) to 100 mm (very active).

Two sub-scales were included in the Hospital Anxiety and Depression Scale (HADS), which was used for measuring anxiety and depression over the prior month (29). Seven items could be found in each sub-scale. This evaluation tool has been used in large-scale studies. The Cronbach's alpha value of the questionnaire was 0.850, while the intraclass connection coefficient was 0.900.

The Fatigue Severity Scale questionnaire was used to assess fatigue severity (FSS) (30). It examined nine items; the average of all items served as the overall score, with the higher scores, indicating greater or severe fatigue. This tool has high reliability, high sensitivity, and internal consistency in fatigue evaluation. The Cronbach's alpha value of the questionnaire was 0.852.

The quality of sleep was assessed using the Pittsburgh Sleep Quality Index (PSQI) (31). The Pittsburgh Sleep Quality Index consisted of 19 questions, which included seven aspects. Each aspect can have a score of 0 (no difficulty) to 3 (severe difficulty), with the total score ranging from 0 to 21. The Cronbach's alpha value of the questionnaire was 0.796.

The Short Form 36 Health Survey (SF-36) was used to assess QoL (32). It evaluated eight aspects. The scores ranged from 0 to 100, with higher scores indicating better health status. There were two forms of scores: the Z-transformed scores and the normalized domains scores. They were divided into physical components summary (PCS) and mental components summary (MCS) scores, with higher scores indicating better health status. The Chinese version of SF-36 has a Cronbach's alpha of 0.720 to 0.880.

The Global Pain Scale (GPS) includes 20 items related to participants' chronic pain experience (33). Participants indicated their responses on an 11-point scale (from 0 to 10). There were four subscales assessing pain, feelings, clinical outcomes, and activities. For the pain subscale, participants indicated the degree of pain felt currently along with their best, worst, and average pain during the last week, as well as whether they have felt less pain in the last week. The total Cronbach's alpha coefficient was 0.984.

Social Support Rating Scale (SSRS) (34), the Chinese version of SSRS, developed by Xiao Shuiyuan in 1994, was used to identify the social support status. SSRS, which consists of 10 items and three dimensions, was selected for its proven reliability and validity. The Cronbach's alpha of the total scale and subscales ranged from 0.825 to 0.896.

### Statistical Analysis

SPSS version 25.0 (IBM, Armonk, NY, USA) was used for the statistical analysis. For measurement data, we first performed a normality test. If the data were normally distributed, measurement data were expressed by using means and standard deviations (SDs); if the data were not normally distributed, then the measurement data were expressed by the median and interquartile range; for categorical data, rates or composition ratios were used. R language (Vienna, Austria) was used to deal with missing values, using the Mice package (Multiple Imputation).

The SEM of SPSS version 25.0 (IBM, Armonk, NY, USA) was used for path analysis. The parameters of the model were estimated by the maximum likelihood method. First, the initial path model was adjusted based on two criteria. One was to delete insignificant paths, and the other was to use the modification index to establish the correlation between some residuals using the combination of professional knowledge to gain the best model.

## Results

A total of 603 patients with RA were consecutively invited to participate, and 580 (96.2%) were eventually included in the present study: 255 (44.0%) from Nantong, 120 (20.7%) from Henan, 101 (17.4%) from Suzhou, 55 (9.5%) from Changzhou, and 49 (8.4%) from Shanghai.

There were 603 questionnaires in total, of which 23 were considered invalid (e.g., with missing answers, highly similar options). The response rate of the questionnaire was 96.2% (580/603) and the proportion of missing values for basic information was 0.7%, with missing information in area (1/580), age (2/580), and height (1/580); the proportion of missing values for other variables were as follows: BID 0.5% (3/580); social support 0.9% (5/580); sleep 1.0% (6/580); pain 0.5% (3/580); medication compliance 0.3% (2/580); and QoL 0.7% (4/580).

### Characteristics of Patients

Table 1 shows the participants' baseline characteristics. The mean age of participants was 51.04 years (SD = 24.65), and 88.4% were female. Overall, 49.3% lived in a suburb. Most patients (91.2%) were married. Only 13.8% of the patients received education for ≤ 9 years. Most (72.2%) were employed, and 31.7% had yearly per capita incomes of <15,000 RMB. The mean disease duration was 4 years. Approximately 14.7% of patients had hypertension, 5% had diabetes, 6% had coronary heart disease, 3.8% had nephropathy, and 12.1% had another cardiopulmonary disease.

**Table 1:** Characteristics of patients with RA (*n* = 580).

**Variables**	***N*/Mean/Median**	**%/SD/IQR**
**Gender**		
Male	67	11.6%
Female	513	88.4%
Age (years)	51.04	24.65
BMI (kg/m^2^)	22.36	4.07
**Location**		
City	132	22.8%
Town	162	27.9%
Suburb	286	49.3%
**Marital status**		
Married	529	91.2%
Unmarried	26	4.5%
Other	25	4.3%
**Education**		
≤ 9 years	80	13.8%
9–12 years	388	66.9%
>12 years	112	19.3%
**Work status**		
Employed	419	72.2%
Unemployed	81	14.0%
Student	5	0.9%
Other	75	12.9%
**Yearly income (RMB)**		
<15,000	184	31.7%
15,000–33,000	218	37.6%
>33,000	178	30.7%
**Smoker**		
Yes	69	11.9%
No	511	88.1%
**Alcohol use**		
Yes	90	15.5%
No	490	84.5%
Disease duration	4.0	(2.0, 9.0)
**Hypertension**		
Yes	85	14.7%
No	495	85.3%
**Diabetes**		
Yes	29	5%
No	551	95%
**Coronary heart disease**		
Yes	35	6%
No	545	94%
**Nephropathy**		
Yes	22	3.8%
No	558	96.2%
**Other cardiopulmonary disease**		
Yes	70	12.1%
No	510	87.9%
**To be hospitalized**		
Yes	206	35.6%
No	374	64.4%
Exercise frequency (/week)	0	(0.2)
Exercise duration (min)	0	(0.10)

*IQR, interquartile range; RA, rheumatoid arthritis; RMB, renminbi; SD, standard deviation*.

### Total Scores of Scales

The mean (SD) of anxiety and depression scores were 10.67 (2.38) and 10.01 (2.39), respectively. The mean (SD) for each scale was 38.92 (7.35) for Fatigue Severity Scale, 26.00 (14.83) for BIDQ, 5.00 (1.50) for DAS28, 10.47(3.01) for PSQI, 55.91 (17.70) for Global Pain Scale, 177.26 (62.19) for PCS, 217.8 (64.63) for MCS, 436.57 (127.02) for the SF-36, and 37.48 (5.34) for SSRS.

### Subgroup Differences

In Table 2, the PCS, MCS, and SF-36 total scores in our study were presented according to the sociodemographic and characteristics of the patients.

**Table 2:** Subgroup comparisons of 36-item short form survey physical component scores, mental component scores, and total scores.

**Characteristic**	**PCS**			**MCS**			**SF-36**		
	***M***	**(SD)**	***P***	***M***	**(SD)**	***P***	***M***	**(SD)**	***P***
**Gender**									
Male	167.39	45.11	0.074	200.73	39.88	0.001^**^	408.79	92.58	0.015^*^
Female	178.55	64		220.03	66.9		440.2	130.48	
**Age (y)**									
18–34	194.23	61.42	<0.001^***^	238.51	64.54	<0.001^***^	475.6	124.8	<0.001^**^
35–59	178.71	63.77		213.6	67.04		434.17	132.76	
≥60	153.02	51.43		201.5	52		393.56	99.84	
**Location**									
City	179.27	56.3	0.129	215.36	55.2	0.005^**^	440.28	110.4	0.028^*^
Town	184.38	63.63		231.59	70.9		456.47	136.33	
Suburb	172.29	63.7		211.12	63.95		423.53	127.55	
**Marital status**									
Married	191.69	64.45	0.305	231.36	57.71	0.508	464.4	121.81	0.453
Unmarried	176.03	62.89		216.93	66.06		434.64	129.52	
Other	188.2	39.66		222.22	32.46		448.42	59.98	
**Education**									
≤ 9 years	150.61	43.72	<0.001^***^	196.3	48.16	<0.001^***^	387.54	85.76	<0.001^***^
9–12 years	175.93	63.83		216.24	67.55		433.01	133.08	
>12 years	200.91	59.37		238.58	58.73		483.91	114.59	
**Work status**									
Employed	181.21	58.78	0.06	221.57	62.68	0.03^*^	443.3	122.57	0.15
Unemployed	162.67	76		198.49	70.89		408.99	144.66	
Student	190.8	72.7		232.17	75.55		442.97	136.77	
Other	170.04	61.83		216.64	65.14		428.35	128.73	
**Yearly income (RMB)**									
<15,000	164.55	68.92	<0.001^***^	209.36	75.61	0.02^*^	412.37	144.06	0.001^**^
15,000–33,000	177.06	56.24		216.51	56.69		434.62	114.75	
>33,000	190.63	59.27		228.11	60.25		463.97	117.51	
**Smoker**									
Yes	176.19	34.89	0.879	210.84	36.09	0.341	427.61	63.84	0.533
No	177.4	65.02		218.74	67.53		437.78	133.27	
**Alcohol use**									
Yes	180.13	49.57	0.634	214.17	51.48	0.562	435.14	97.79	0.907
No	176.73	64.26		218.8	66.79		436.83	131.76	
**Disease duration**									
≤ 1	175.02	68.23	<0.001^***^	220.65	78.39	0.62	428.42	143.92	0.017^*^
1–3	190.74	61.46		221.22	59.69		456.06	121.9	
3–5	189.18	53.11		220.08	54.75		455.13	109.35	
≥5	164.43	61.47		213.42	65.4		419.52	128.01	
**Comorbidities**									
No	180.21	65.23	0.122	223.09	70.56	0.008^**^	445.11	137.02	0.028^*^
Yes	171.85	55.94		208.12	50.82		420.95	104.84	
**Exercise**									
No	175.55	65.25	0.387	216.28	68.43	0.46	433.7	134.37	0.478
Yes	180.18	56.6		220.8	64.63		441.47	113.47	
**BMI**									
<18.5 kg/m^2^	175.56	53.79	0.966	213.66	62.79	0.311	431.95	117.09	0.651
18.5–23.9 kg/m^2^	177.7	59.92		220.77	62.52		440.09	121.77	
≥24 kg/m^2^	176.75	70.73		211.67	70.38		429.17	143.4	

*BMI, body mass index; MCS, mental component score; PCS, physical component score; RMB, renminbi; SF-36, The Short Form 36 health survey*.

* * < 0.05*,

** *< 0.01, and*

*** *< 0.001*.

### Correlations

The pain score had a significant correlation with DAS28, fatigue, sleep, and body image (*p* < 0.01). Body image score had a significant correlation with DAS28, depression, fatigue, sleep (*p* < 0.01). Sleep score had a significant correlation with depression, fatigue (*p* < 0.01).

### Structural Equation Model

**Figure 2:**
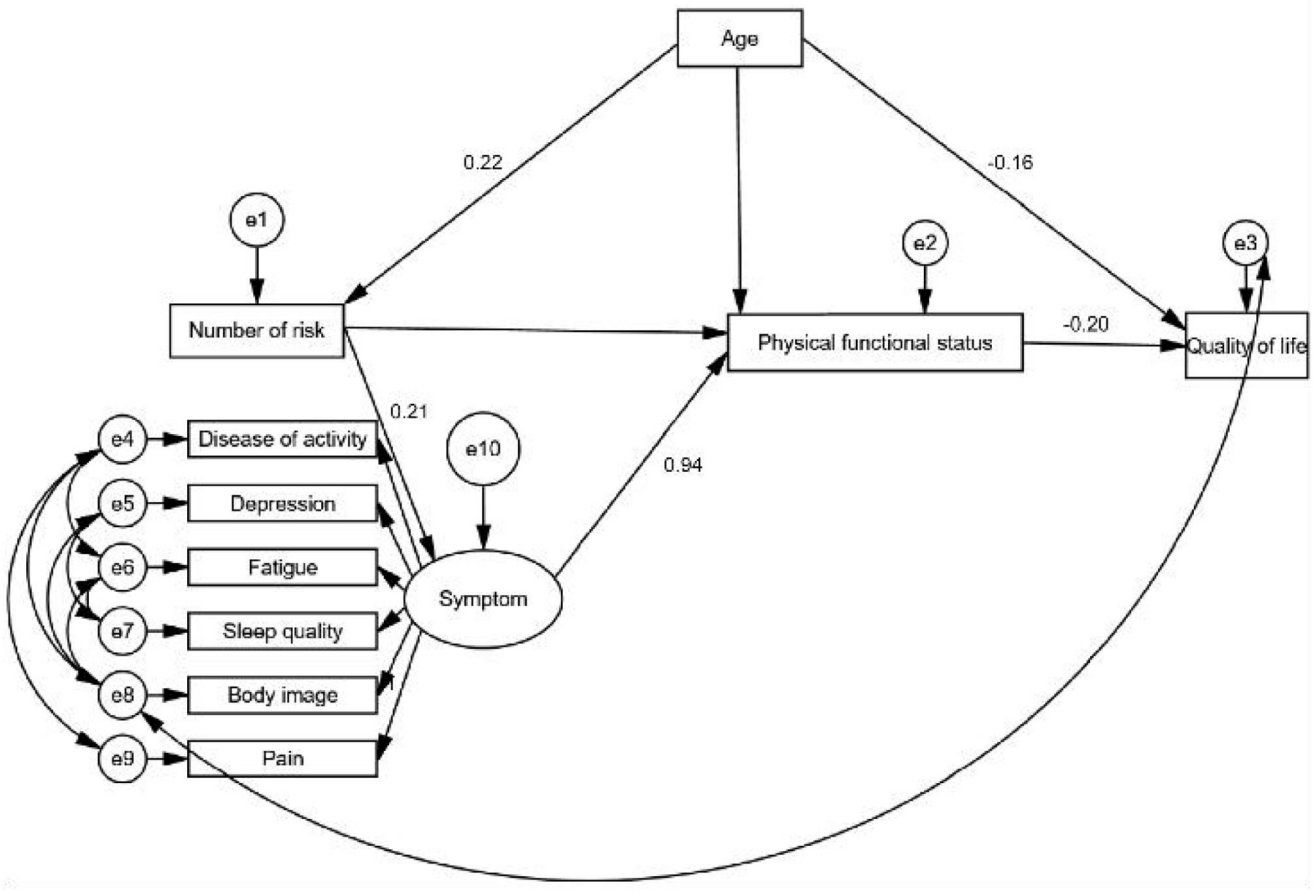
Goodness of fit of structural equation models of variables in a cross-sectional study of health-related quality of life. (1) Latent variable: biological and physiological status; measured variables: number of risk factors (e.g., hyperlipidemia, diabetes). (2) Latent variable: symptom status; measured variable: BIDQ, DAS28, HADS, FSS, PSQI, and GPS. BIDQ, Body Image Disturbance Questionnaire; DAS28, Disease Activity Score in 28 joints; HADS, Hospital Anxiety and Depression Scale; FSS, Fatigue Severity Scale questionnaire; PSQI, Pittsburgh Sleep Quality Index; GPS, Global Pain Scale. (3) Latent variable: physical functional status; measured variable: physical function status was based on the total score of “go to the store,” “do chores in my home,” “enjoy my friends and family,” “exercise,” and “participate in my favorite hobbies” in GPS. GPS, Global Pain Scale. (4) Latent variable: QoL; measured variable: SF-36 total score. QoL, quality of life; SF-36, Short Form 36 Health Survey. (5) Latent variable: characteristics of the environment; measured variable: social support. (6) Latent variable: characteristics of the individual measured variable: demographic characteristics (age, education, income, etc).

Figure 2 shows significant pathways in the final HRQoL model. We have successively removed the unimportant paths. Modification indices indicated no modifications. Figure 2 shows the final model. The indices of the goodness-of-fit showed that the final model was an excellent fit to the data with root mean square error of approximation (RMSEA) of 0.072, goodness-of-fit index of 0.968, adjusted goodness of fit index of 0.928, normed fit index of 0.955, relative fit index of 0.916, incremental fit index of 0.936, and comparative fit index of 0.966. The direct, indirect, as well as the overall effects of predictors on HRQoL are demonstrated in Figure 2. Age and PF had an indirect effect on HRQoL. Among symptoms that had indirect effects on HRQoL through the physical function status, age exerted a direct influence on HRQoL and an indirect influence on HRQoL via PF. The direct effect value between QoL paths and the path correlation coefficient between symptoms are shown in Figure 2.

Fitting indicators of the model: ratio of chi square/df = 3.960; RMSEA = 0.072; goodness-of-fit index = 0.968; adjusted goodness of fit index = 0.928; normed fit index = 0.955; relative fit index = 0.916; incremental fit index = 0.966; Tacker-Lewis index = 0.936; and comparative fit index = 0.966. The coefficients of related factors were standardized, and the standardized regression coefficients were sorted. Symptoms and the standardized factor load of variables showed that the standardized load of pain was as high as 0.99, which is the most important factor affecting QoL. The standardized load of body image was 0.63, the standardized load of fatigue was 0.25, the standardized load of sleep quality was 0.24, the standardized load of depression was 0.07.

## Discussion

Wilson and Cleary's model-associated factors with global HRQoL environment variables were assessed in this study. This is the first large-sample multi-center cross-sectional survey using multiple variables to investigate the associations between the individual characteristics of patients with RA and overall HRQoL in China using SEM. The final model provided here shows an association between clinical variables, such as other underlying diseases, that mediate an individual's experience with actual symptoms, physical functioning, and general health on HRQoL. SEM allows the simultaneous assessment of the effects of personal and environmental characteristics on potential variables in the model. Our study and previous studies show that patients with RA often experience chronic pain and functional disabilities, including a high incidence of depression, body image disorders, fatigue, and sleep disorders with a decline in patients' QoL (35).

In the analysis of the subgroup, HRQoL scores were higher (indicating better QoL) in men than in women, the employed than in the unemployed, the college-educated individuals than in those with less educational levels, and in those in third-highest rank of income (¥15,000). The marital statuses were not associated with the HRQoL overall score, PCS, or MCS. These results are consistent with Gong's study findings and are worthy of attention (36). There were also significant differences in the QoL of patients with RA in different subgroups of age, disease duration, and comorbidities, which is consistent with Zeng's et al.'s study (35).

The presence of comorbidities may increase mortality in patients with RA. Further, treatment strategies may be affected, leading to worsening conditions. Fitting the SEM of the QoL of patients with RA in our study showed similar results for latent variables of biological factors, and both the number of comorbidities and risks, including coronary heart disease, diabetes, kidney disease, and fractures. Accordingly, the prediction and management of comorbidities are increasingly important in the long-term management of RA (37). What should not be ignored is that age as an individual characteristic not only had a constant effect on physical functioning but also on HRQoL. These data provide information about the prevalence, incidence, risk factors, and other characteristics of selected comorbidities, which may help identify comorbidities and management strategies.

Joint pain is often the first symptom. The definition of pain has been revised to “An unpleasant sensory and emotional experience associated with the diagnosis, treatment as well as rehabilitation of RA shows a long-term, dynamic together with complicated pragmatic procedure with, or resembling that associated with, actual or potential tissue damage” (38). In addition, there is an involvement of sensory perception, which is implicated in emotional processes and negative outcomes. Importantly, the unique sensory processing patterns of individuals have been reported as crucial factors in determining negative outcomes in the clinical practice and play a role in the prediction of their QoL (39). In our study, in order to fully evaluate the multidimensional and complex pain of RA patients, pain assessment was performed using Global Pain Scale; this is the first time that a comprehensive pain assessment was applied to RA patients. It is also an innovative approach used in this study. In the SEM results, a better fit may also better reflect the patient's complications, in terms of pain, feelings, outcomes, and activities. Chronic disease, such as RA, is characterized by its uncertain course, and its frequent confrontation with pain and fatigue, and the possibility of becoming disabled influence the patients' psychological well-being. Notably, the results of our Pearson's correlation analysis showed that pain correlates with disease activity, fatigue, sleep quality, and physical image disorders, and in the results of the SEM, the standardized load of pain is up to 0.99, which is sufficient to prove that pain plays a decisive role in QoL. This suggests that we should pay attention to the pain symptoms and related symptom groups, strengthen the symptom management of RA patients, and improve the QoL of RA patients.

Disease activity was assessed by using the DAS28. Our results showed that the DAS28 score was 5.00 (1.50), which was more severe than the results of previous research (40), SEM shows that disease activity interacts with fatigue, BID, and pain, and affects body function. These results are completely consistent with the results of the Pearson's correlation analysis.

In patients with RA, fatigue is considered the most common extra-articular symptom other than pain. Among patients with RA. Our study found that 64.8% of patients considered fatigue the most important issue. In the correlation analysis, fatigue was associated with pain, BID, and sleep quality. In SEM, it was also shown to interact with increased disease activity, with a standardized load factor of 0.25, ranking fourth among the symptom groups. This is similar to the findings of Gong's study (36). Pain is the dominant factor in the experience and degree of fatigue. Disease activity is positively correlated to fatigue. Thus, fatigue has been shown to cause notable adverse consequences and to affect every aspect of daily life, bringing a considerable human and economic burden to QoL, thereby reducing the overall health of patients (41).

Psychological well-being refers to an individual's mood in a global sense. When someone is confronted with uncertainty, threat, and ambiguity, this may provoke feelings of depression. Our results show that 76.4% of patients with RA experienced depression. Patients with RA and depression had significantly lower medication compliance, impaired physical function, sleep quality, and QoL than those without depression (42). Sleep disturbances can be often observed in patients who have long-term diseases; meanwhile, the prevalence of poor sleep is higher in patients with RA than in those without RA.

Body image (BI) is defined as “the attitudes and perceptions of individuals toward their appearance and their beliefs and others with respect to their body.” It is strongly influenced by one's health and may be associated with abnormal coping behavior, psychopathology, poor outcomes, and HRQoL (43). As RA progresses, some patients may have irreversible damage, such as joint deformities, reduced restrictions on movement, and function, which may lead to psychological problems, such as human BID (44). Our results showed that almost all patients observe their BI and have different degrees of BID. In the correlation analysis, BID was associated with disease activity, fatigue, depression, sleep quality, pain, and, interestingly, SEM. In addition to indirect effects on QoL, BI directly affects the QoL. The standard factor load is 0.63, which plays a decisive role in pain. It is likely to be a mediating effect in the RA QoL model. The important conclusion of the study is also the innovation of this study, which deserves more attention from rheumatologists.

The QoL of patients with RA is a complex systemic response, which is determined by the patient's biological characteristics. There are different multivariable symptom group interactions, and it is not possible to simply explore the correlations of several variables. A systematic study is needed, and important factors should be assessed by using multi-factor analysis and SEM to verify its causality. In our research hypothesis, social support played a role in the QoL model as an environmental feature but it was included in the final model verification, differing from the results of another study on the QoL of patients with RA (45). It may be that their research is more related to the factorial analysis of its relevance to the social quality, but it is similar to Gong's results (36). Social support is a long-term process, associated with factors, such as the route and frequency of social support, and patient demand. This suggests that we should conduct a comprehensive and dynamic evaluation of the entire process in the patient support system, as needed while paying attention to the selected methods and durations to improve the QoL of patients with RA.

There are some limitations to this study. First, self-reporting was used to assess the patient's condition. It is difficult to avoid recall and reporting biases, which may have affected the association between variables. Second, this was a multi-center cross-sectional survey. Future research should focus longitudinally on the QoL of patients with RA and explore intermediary factors affecting their QoL, Qualitative research will be combined with patient interviews to focus on the vertical regularity of the deterioration of QoL, the needs, cognition, and experience in the course of the disease. We should also pay attention to the influence of environmental factors, coping style, and symptom management on patients' quality of life to provide better evidence to establish effective interventions.

In conclusion, this study analyzed the QoL of patients with RA in China through a multi-center survey. The QoL model was used to fit an SEM to systematically verify and analyze the population disease data, biological factors, and the direct and indirect effects of the symptom group on the QoL, and the interactions between the symptoms. Our results showed that age and comorbidities would directly influence QoL, pain, BI, disease activity, fatigue, sleep quality as and depression, which are ranked according to whether the effects are important to the patient's physical function and on the patient's QoL. BI has a direct impact on QoL. Therefore, the RA diagnosis and treatment and rehabilitation of RA patients is a long-term, dynamic and complex practical process. Patients' personal symptoms, needs, and experiences also vary greatly. Comprehensive assessment of patients' symptoms, needs, and experiences, at the same time, the role of social support cannot be ignored, as they can help to meet patients' nursing needs, improve their mood and pain-based symptom management, and ultimately improve patients' QoL.

## Data Availability Statement

The original contributions presented in the study are included in the article/supplementary files, further inquiries can be directed to the corresponding authors.

## Ethics Statement

The studies involving human participants were reviewed and approved by The Ethics Committee of Soochow University. The patients/participants provided their written informed consent to participate in this study.

## Author Contributions

BS and HC performed the experiment, analyzed the data, and prepared the figures and the manuscript. BS, HC, AD, RX, YG, and XC collected the data. DY analyzed the data. G-YX, HL, OY, and CY designed, supervised the experiments, and finalized the manuscript. All the authors have read and approved the manuscript.

## Conflict of Interest

The authors declare that the research was conducted in the absence of any commercial or financial relationships that could be construed as a potential conflict of interest.

## Publisher's Note

All claims expressed in this article are solely those of the authors and do not necessarily represent those of their affiliated organizations, or those of the publisher, the editors and the reviewers. Any product that may be evaluated in this article, or claim that may be made by its manufacturer, is not guaranteed or endorsed by the publisher.

## Abbreviations

QoL: quality of life
SEM: structural equation modeling
RMSEA: root mean square error of approximation
HRQoL: health-related quality of life
RA: rheumatoid arthritis
BID: body image disturbance
DAS28: Disease Activity Score in 28 joints
SF-36: Short Form 36 Health Survey
PCS: physical components summary
MCS: mental components summary
SSRS: Social Support Rating Scale
SD: standard deviation
IQR: interquartile range
RMB: renminbi
BMI: body mass index.
